# Intraventricular synchronism assessment by gated-SPECT myocardial perfusion imaging in cardiac resynchronization therapy. Does cardiomyopathy type influence results?

**DOI:** 10.1186/s13550-020-00703-4

**Published:** 2020-10-20

**Authors:** Amalia Peix, Kenia Padrón, Lázaro O. Cabrera, Osmín Castañeda, Danet Milán, Jesús Castro, Roylan Falcón, Frank Martínez, Lydia Rodríguez, Jesús Sánchez, Erick Mena, Regla Carrillo, Yoel Fernández, Ricardo Escarano, Diana Páez, Maurizio Dondi

**Affiliations:** 1 Nuclear Medicine Department, Institute of Cardiology and Cardiovascular Surgery, 17 #702, Vedado, 10 400, La Habana, Cuba; 2 Nuclear Medicine and Diagnostic Imaging Section, Division of Human Health, International Atomic Energy Agency, Vienna, Austria

**Keywords:** Non-compaction myocardium, Cardiomyopathies, Cardiac resynchronization therapy, Gated-SPECT, Phase analysis, Intraventricular dyssynchrony

## Abstract

**Purpose:**

To analyze the evolution post-cardiac resynchronization therapy (CRT) in left ventricular non-compaction (LVNC) cardiomyopathy (CM) patients compared to other types of CM, according to clinical and functional variables, by using gated-SPECT myocardial perfusion imaging (MPI).

**Methods:**

Ninety-three patients (60 ± 11 years, 28% women) referred for pre-CRT assessment were studied and divided into three groups: 1 (non-ischemic CM with LVNC, 11 patients), 2 (ischemic CM, 28 patients), and 3 (non-ischemic CM, 53 patients). All were studied by a ^99m^Tc-MIBI gated-SPECT MPI at rest pre-CRT implantation and 6 ± 1 months after, including intraventricular dyssynchrony assessment by phase analysis. Quality of life was measured by the Minnesota Living with Heart Failure Questionnaire (MLHFQ).

**Results:**

No differences in sex, atherosclerotic risk factors other than smoking habit, and MLHFQ results were found among groups. LVNC CM patients were younger, with greater QRS width and lower left ventricular ejection fraction (LVEF) at baseline, but the differences were not significant. No significant differences were found at baseline regarding ventricular function, although end-systolic volume was slightly higher in LVNC CM patients. Mean SRS was significantly higher (*p* < 0.0001) in ischemic patients (14.9) versus non-ischemic ones (8.7 in group 1 and 9 in group 2). At baseline, LVNC CM patients were significantly more dyssynchronous: Their phase standard deviation (PSD) was higher (89.5° ± 14.2°) versus groups 2 (65.2° ± 23.3°) and 3 (69.7° ± 21.7°), *p* = 0.007. Although the quality of life significantly improved in all groups, non-ischemic patients (with or without LVNC) showed a higher LVEF increase and volumes reduction at 6 months post-CRT. Dyssynchrony reduced post-CRT in all groups. Nevertheless, those more dyssynchronous at baseline (LVNC CM) exhibited the most significant intraventricular synchronism improvement: PSD was reduced from 89.5° ± 14.2° at baseline to 63.7° ± 20.5° post-CRT (*p* = 0.028). Six months post-CRT, 89% of patients were responders: 11 (100%) of those with LVNC CM, 25 (86%) of those with ischemic CM, and 47 (89%) of patients with non-ischemic CM. No patient with LVNC CM had adverse events during the follow-up.

**Conclusion:**

CRT contributes to a marked improvement in non-ischemic CM patients with non-compaction myocardium. Phase analysis in gated-SPECT MPI is a valuable tool to assess the response to CRT.

## Introduction

Heart failure (HF) affects more than 15 million people worldwide, causing significant increases in disability, mortality, and healthcare costs [1, 2]. Coronary artery disease is the main cause of HF and accounts for 60–70% of the cases [3].

Isolated left ventricular non-compaction (LVNC) is considered a particular myocardial morphological abnormality [4, 5], which can be diagnosed both in otherwise normal hearts and in association with different types of cardiomyopathy, but it is most commonly found in association with a dilated cardiomyopathy (DCM) [4, 6, 7].

Cardiac resynchronization therapy (CRT) benefits patients with end-stage HF, reduced left ventricular ejection fraction (LVEF) (< 35%), and a wide QRS complex on electrocardiogram (> 120 ms) [8]. Nevertheless, it has been reported that 20 to 40% of patients did not benefit from CRT according to these criteria [9, 10]. Electrical dyssynchrony as determined by QRS duration may not inevitably represent real mechanical dyssynchrony and, therefore, is not the best predictor of CRT response [11, 12]. Thus, assessment of cardiac mechanical dyssynchrony represents a better option to more accurately select patients who would benefit from CRT [13, 14].

LV mechanical dyssynchrony has been assessed by different imaging techniques, such as echocardiography with tissue Doppler imaging, strain imaging, and more recently speckle tracking; magnetic resonance imaging; gated blood pool ventriculography; and gated single-photon emission computed tomography (gated-SPECT) [15–19]. However, among all of these techniques, gated-SPECT myocardial perfusion imaging (MPI) is the only one able to give, in a reproducible way, information about global and regional ventricular function, presence of intraventricular synchronism, and myocardial perfusion with the same test. Phase analysis of gated-SPECT MPI has been applied to the evaluation of mechanical intraventricular synchronism [19, 20], and phase histogram standard deviation (PSD) and histogram bandwidth (HBW) have been recognized as the most important indices of intraventricular synchronism [21].

Some of the patients included in the present study are part of the International Atomic Energy Agency (IAEA)-sponsored non-randomized, multicenter trial: “Value of intraventricular synchronism assessment by gated-SPECT myocardial perfusion imaging in the management of heart failure patients submitted to cardiac resynchronization therapy” (IAEA VISION-CRT). In this trial, 195 consecutive patients from 8 countries were enrolled. All underwent gated-SPECT MPI before and 6 months after CRT, and it was concluded that LV dyssynchrony improvement by gated-SPECT MPI, but not on-target lead placement, predicted clinical outcomes in patients undergoing CRT [22]. In addition, other papers showing the association of septal thickening improvement with reverse remodeling, improvement in LVEF, and reduction of left ventricular dyssynchrony [23], as well as the favorable effect of CRT on LV diastolic dyssynchrony [24] have been also published. Recently, another paper was published on the technical aspects of gated-SPECT MPI assessment of left ventricular dyssynchrony used in the VISION-CRT study [25].

But thus far, little is known about the effects of CRT in LVNC CM patients [26–28]. In particular, whether the benefits of CRT in LVNC CM are similar to other types of CM (ischemic and non-ischemic). In addition, as far as we know, the imaging technique used to functionally assess these patients was always echo. Thus, the aim of the present work was to analyze the evolution post-CRT in LVNC CM patients compared to other types of cardiomyopathies, according to clinical and functional variables, by using gated-SPECT MPI.

## Material and methods

### Study population

We studied 93 patients (mean age: 60 ± 11 years, 28% women) who were referred to the Nuclear Medicine Department of the Institute of Cardiology and Cardiovascular Surgery from April 2014 to April 2017 with the following inclusion criteria: males and females older than 18 years of age, with New York Heart Association (NYHA) functional class II, III, or ambulatory IV for at least three months before enrolment, despite receiving optimal tolerated medical therapy according to current guidelines, LVEF ≤ 35% from ischemic or non-ischemic causes, intrinsic QRS duration of ≥ 120 ms, with morphology of left bundle branch block (LBBB), and sinus rhythm. Exclusion criteria were as follows: arrhythmias that prevented gated acquisition; major coexisting illness affecting survival less than 1 year; right bundle branch block; pregnancy or breastfeeding; acute coronary syndromes, coronary artery bypass grafting, or percutaneous coronary intervention in the last three months before enrolment and within six months of CRT implantation.

All patients were studied by technetium-99 m methoxy-isobutyl-isonitrile (^99m^Tc-MIBI) gated-SPECT MPI at rest within up to four weeks before the CRT implantation and 6 ± 1 months after. In addition, they were monitored by consultations and telephone calls (to patients or next of kin) at 1, 3, 6, 12, and 24 months. All 93 patients underwent CRT and gated-SPECT both at baseline and at six months, including clinical six-month follow-up data, but complete data of clinical assessment at 2 years were completed in 81 patients.

NYHA class was assessed by a clinical cardiologist unaware of the imaging results as determined by the core laboratory. Minnesota Living with Heart Failure Questionnaire (MLHFQ) was administered by study personnel, and change was assessed by the conventional five-point criteria.

Improved clinical response was defined as at least one of the following 3 points at six months: improvement of LVEF by ≥ 5%, reduction of end-systolic volume (ESV) by ≥ 15%, and improvement by at least 5 points in MLHFQ.

Patients were divided into three groups: 1 (non-ischemic CM with non-compaction myocardium, 11 patients, 12%), 2 (ischemic CM, 28 patients, 30.4%), and 3 (non-ischemic CM, 53 patients, 57.6%). Patients were considered as ischemic if they had a previous history of myocardial infarction, other acute coronary syndromes, or ischemia demonstrated in a noninvasive imaging test (echo-stress or mainly gated-SPECT MPI). As all these patients were studied by echo before inclusion in the study, those who showed signs of non-compaction left ventricular myocardium by echo [29] composed the group 1. Some of the patients included in the present study are part of a coordinated research project sponsored by International Atomic Energy Agency (IAEA): “Value of intraventricular synchronism assessment by gated-SPECT myocardial perfusion imaging in the management of heart failure patients submitted to cardiac resynchronization therapy.”

The present study complies with the ethical standards laid down in the 1964 Declaration of Helsinki and all subsequent revisions. The review board and Ethics Committee of the Institute of Cardiology approved the study, written informed consent was obtained from all patients prior to the inclusion in the study, and patient anonymity was maintained during data analysis.

### Gated-SPECT MPI acquisition and interpretation

Rest images were acquired between 30 and 60 min after the intravenous injection of 15 mCi of ^99m^Tc-MIBI using a rotating dual-head gamma camera (Nucline Spirit DHV, Mediso, Hungary) equipped with low-energy, high-resolution, parallel-hole collimators, with a 20% energy window centered on the 140 keV photopeak. Sixty-four projections (20 s per projection), eight frames/cycle, with a 64 × 64 matrix were obtained over a 180°′ orbit. Imaging was always performed in a supine position.

All images were reconstructed using OSEM with 3 iterations and 10 subsets and filtered by a Butterworth filter, power 10, using a cutoff frequency of 0.3 cycles/mm. No attenuation or scatter correction was applied.

Semi-quantitative visual interpretation of images employed short-axis and vertical long-axis tomograms divided into 17 segments [30]. Each segment was scored by the consensus of two expert independent observers who were unaware of the clinical and angiographic data, using a five-point scoring system (from 0 = normal to 4 = absence of myocardial uptake). Disagreements, including any score in each SPECT segment, were resolved by consensus. Summed rest scores (SRS) were obtained to assess perfusion at rest.

An operator independent analysis of LVEF and ventricular volumes was made using dedicated software (Emory Cardiac Toolbox –ECTb-, Syntermed, Inc., Atlanta, Georgia, USA). The left intraventricular mechanical dyssynchrony was evaluated by using the phase analysis of the gated-SPECT MPI included in the ECTb, previously described [19]. Left ventricular dyssynchrony was defined as left ventricular PSD > 43° [31].

### Statistical analysis

Categorical variables are expressed as numbers and percentages and compared when necessary with the Chi-square test and the Fisher exact test. Continuous variables are expressed as mean ± standard deviation (SD), and the nonparametric Kolmogorov–Smirnov normality test (K–S test) was applied to check variables normality. For comparison of pre- and post-CRT continuous variables in each group, the paired Student's t test or the Wilcoxon signed-rank test were applied. One-way ANOVA was used for comparison of continuous variables among groups, including the post hoc Tukey's test when necessary. All analyses were performed using SPSS 15.0 (SPSS Inc., Chicago, IL, USA). A value of *p* < 0.05 was considered statistically significant.

### Results

Clinical characteristics of the 93 patients are shown in Table 1. No differences in sex, atherosclerotic risk factors other than smoking habit, and MLHFQ results were found among groups. Ischemic patients were older (*p* = 0.04) and included more smokers (*p* = 0.021) than the rest of patients. Non-compaction CM patients were younger, with greater mean QRS width and lower LVEF at baseline, although the differences were not significant. CRT-Implantable cardioverter-defibrillator (ICD) (Syncra CRT-ICD) was implanted in the majority of patients (64%, 59%, and 62% of groups 1, 2, and 3, respectively), and the lateral wall was preferred for lead placement in the three groups (82%, 93%, and 91%, respectively). The rest of patients received a Sincra CRT pacing.

**Table 1:** Characteristics of Patients

Variables	Non-compaction (*n* = 11)	Ischemic CM (*n* = 29)	Non-ischemic CM (*n* = 53)	*p*
Sex (Men/Women)	6/5	21/8	40/13	NS
Mean Age (years)	57.5 ± 7.8	64.6 ± 9.4	59.1 ± 11.1	0.04
Mean QRS duration (milliseconds)	172.5 ± 32.3	152.6 ± 24.3	156.4 ± 25.1	NS
Mean LVEF for inclusion (%)	22.3 ± 5.7	26.4 ± 5.2	25.3 ± 6.0	NS
High Blood Pressure	6	20	28	NS
Smoking Habit	2	9	4	0.021
Diabetes Mellitus	3	8	5	NS
Dyslipidemia	0	6	7	NS
Family History of CAD	3	12	7	NS
MLHFQ results	48.3 ± 20.8	41.2 ± 19.8	42.9 ± 17.3	NS

Age, QRS duration, LVEF, and MLHFQ results are expressed as mean ± SD. The rest of variables are presented as the number

*CAD* coronary artery disease, *LVEF* left ventricular ejection fraction, *MLHFQ* Minnesota Living with Heart Failure Questionnaire

Gated-SPECT results are shown in Table 2. No significant differences were found at baseline regarding ventricular function (LVEF and volumes), although end-systolic volume (ESV) was slightly higher in non-compaction CM patients. Mean SRS was significantly higher (*p* < 0.0001) in ischemic patients (14.9) versus non-ischemic ones (8.7 in group 1 and 9 in group 2). At baseline, non-compaction CM patients were significantly more dyssynchronous than the rest: Their PSD was higher (89.5 ± 14.2°) versus groups 2 (65.2 ± 23.3°) and 3 (69.7 ± 21.7°), *p* = 0.007. HBW was also higher in group 1 patients.

**Table 2:** Gated-SPECT Myocardial Perfusion Imaging results

Variables	Non-compaction (*n* = 11)	Ischemic CM (*n* = 29)	Non-ischemic CM (*n* = 53)	*p*
SRS	8.7 ± 4.4	14.9 ± 7.1	9.0 ± 3.9	< 0.0001
LVEF (%)	18.0 ± 6.1	22.3 ± 7.4	22.6 ± 7.6	NS
EDV (ml)	360.6 ± 122.0	386.3 ± 113.9	309.6 ± 134.8	NS
ESV (ml)	299.9 ± 119.0	226.6 ± 100.0	246.1 ± 120.1	NS
SV (ml)	62.4 ± 18.3	63.2 ± 31.2	66.0 ± 27.2	NS
PSD (°)	89.5 ± 14.2	65.2 ± 23.3	69.7 ± 21.7	0.007
HBW (°)	247.9 ± 58.6	205.7 ± 62.1	223.9 ± 69.1	NS

Variables are expressed as mean ± SD

*EDV* end-diastolic volume, *ESV* end-systolic volume, *HBW* histogram bandwidth, *LVEF* left ventricular ejection fraction, *PSD* phase standard deviation, *SRS* summed rest score, *SV* systolic volume

Tables 3, 4, and 5 show the comparison between baseline and post-CRT six months’ results in groups 1, 2, and 3, respectively. Although the quality of life significantly improved in all groups, non-ischemic patients (with or without non-compaction) showed a better functional improvement (as by LVEF increase and volumes reduction) at 6 months post-CRT. Patients more dyssynchronous at baseline (those with non-compaction CM) exhibited the more significant improvement on intraventricular synchronism: PSD was reduced from 89.5 ± 14.2° at baseline to 63.7 ± 20.5° post-CRT (*p* = 0.028).

**Table 3:** Comparison between baseline and 6-month follow-up in non-compaction cardiomyopathy patients (Group 1)

Variables	Baseline	Post-CRT	*p*
MLHFQ results	48.3 ± 20.8	16.0 ± 19	0.008
SRS	8.7 ± 4.4	10.0 ± 5.1	NS
LVEF (%)	18.0 ± 6.1	24.7 ± 9.7	0.038
EDV (ml)	360.6 ± 122.0	328.1 ± 125.4	NS
ESV (ml)	299.9 ± 119.0	257.3 ± 117.9	NS
SV (ml)	62.4 ± 18.3	70.8 ± 23.1	0.038
PSD (°)	89.5 ± 14.2	63.7 ± 20.5	0.028
HBW (°)	247.9 ± 58.6	183.3 ± 60.1	NS

Variables are expressed as mean ± SD

*EDV* end-diastolic volume, *ESV* end-systolic volume, *HBW* histogram bandwidth, *LVEF* left ventricular ejection fraction, *MLHFQ* Minnesota Living with Heart Failure Questionnaire, *PSD* phase standard deviation, *SRS* summed rest score, *SV* systolic volume

**Table 4:** Comparison between baseline and 6-month follow-up in ischemic cardiomyopathy patients (Group 2)

Variables	Baseline	Post-CRT	p
MLHFQ results	41.2 ± 19.8	20.5 ± 18.2	0.001
SRS	14.9 ± 7.1	12.3 ± 6.4	NS
LVEF (%)	22.3 ± 7.4	27.5 ± 11.4	0.008
EDV (ml)	386.3 ± 113.9	278.4 ± 142.3	NS
ESV (ml)	226.6 ± 100.0	217.4 ± 134.1	NS
SV (ml)	63.2 ± 31.2	83.9 ± 77.8	NS
PSD (°)	65.2 ± 23.3	58.5 ± 24.8	NS
HBW (°)	205.7 ± 62.1	178.5 ± 74.3	NS

Variables are expressed as mean ± SD

*EDV* end-diastolic volume, *ESV* end-systolic volume, *HBW* histogram bandwidth, *LVEF* left ventricular ejection fraction, *MLHFQ* Minnesota Living with Heart Failure Questionnaire, *PSD* phase standard deviation, *SRS* summed rest score, *SV* systolic volume

**Table 5:** Comparison between baseline and 6-month follow-up in non-ischemic cardiomyopathy patients (Group 3)

Variables	Baseline	Post-CRT	*p*
MLHFQ results	42.9 ± 17.3	16.0 ± 13.8	< 0.0001
SRS	9.0 ± 3.9	8.1 ± 4.0	NS
LVEF (%)	22.6 ± 7.6	30.7 ± 9.8	< 0.0001
EDV (ml)	309.6 ± 134.8	274.3 ± 131.6	NS
ESV (ml)	246.1 ± 120.1	200.9 ± 17.4	0.005
SV (ml)	66.0 ± 27.2	74.7 ± 23.4	NS
PSD (°)	69.7 ± 21.7	60.6 ± 22.8	NS
HBW (°)	223.9 ± 69.1	193.9 ± 72.6	NS

Variables are expressed as mean ± SD

*EDV* end-diastolic volume, *ESV* end-systolic volume, *HBW* histogram bandwidth, *LVEF* left ventricular ejection fraction, *MLHFQ* Minnesota Living with Heart Failure Questionnaire, *PSD* phase standard deviation, *SRS* summed rest score, *SV* systolic volume

**Fig. 1:**
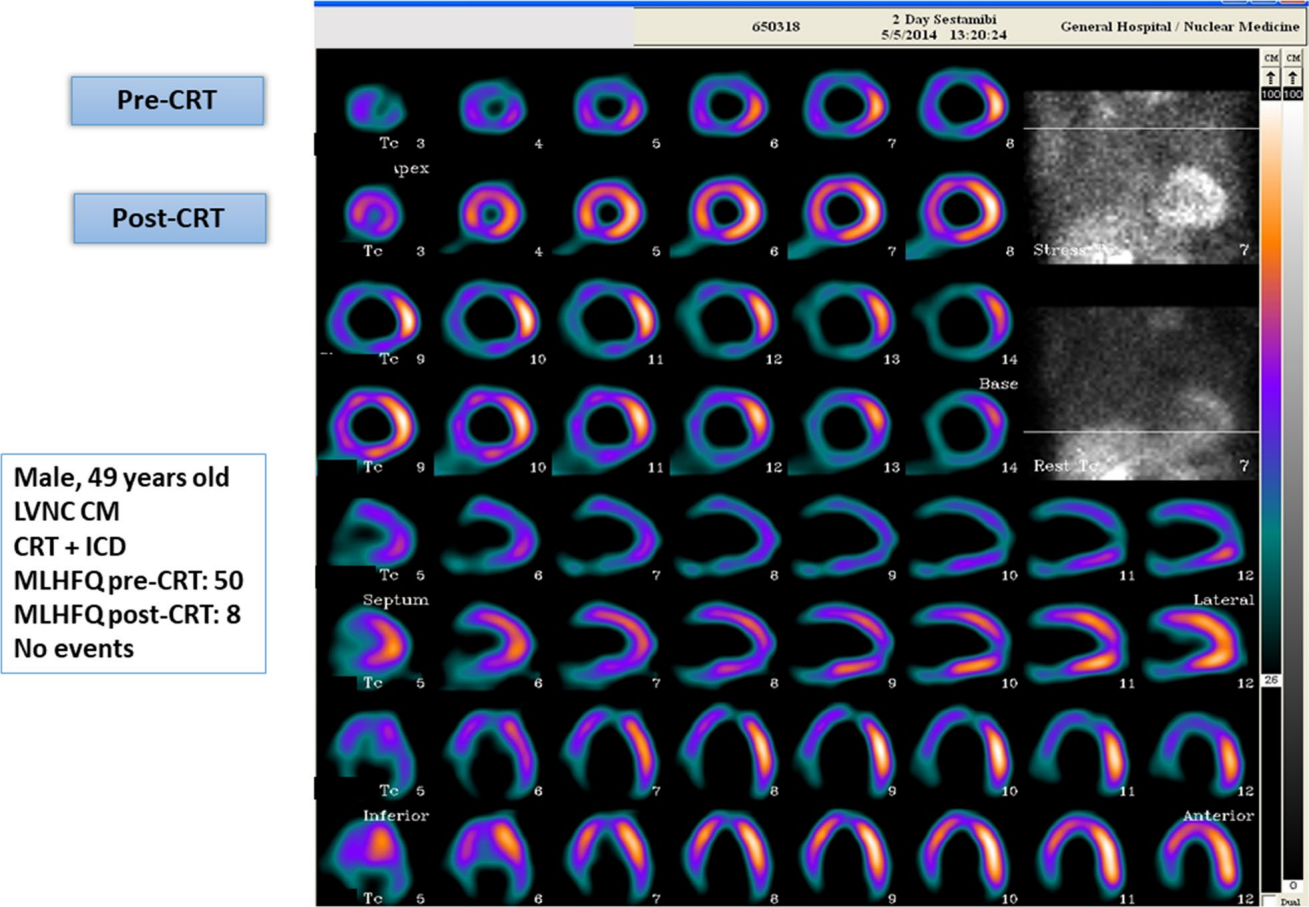
^99^m Technetium-MIBI gated-SPECT MPI in a 49-y.o. male patient with diagnosis of left ventricular non-compaction cardiomyopathy. Perfusion images comparing pre- and post-CRT. *CRT* cardiac resynchronization therapy, *ICD* implantable cardioverter defibrillator, *LVNC CM* left ventricular non-compaction cardiomyopathy, *MLFHQ* Minnesota Living with Heart Failure Questionnaire

**Fig. 2:**
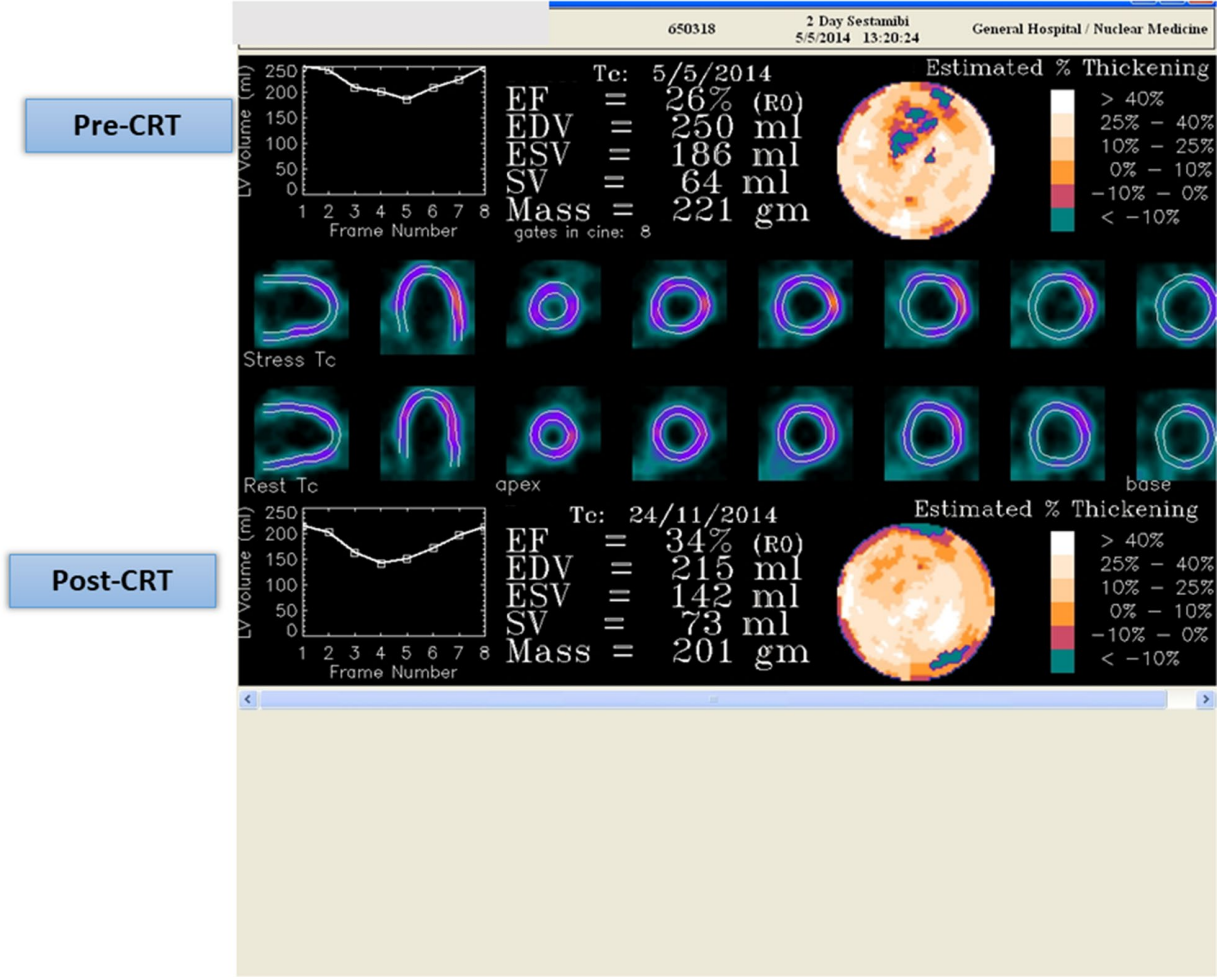
Same patient, ventricular function behavior comparing pre- and post-CRT. There is a functional improvement post-CRT (LVEF increase and ventricular volumes reduction). *CRT* cardiac resynchronization therapy, *EF* ejection fraction, *EDV* end-diastolic volume, *ESV* end-systolic volume, *SV* stroke volume

**Fig. 3:**
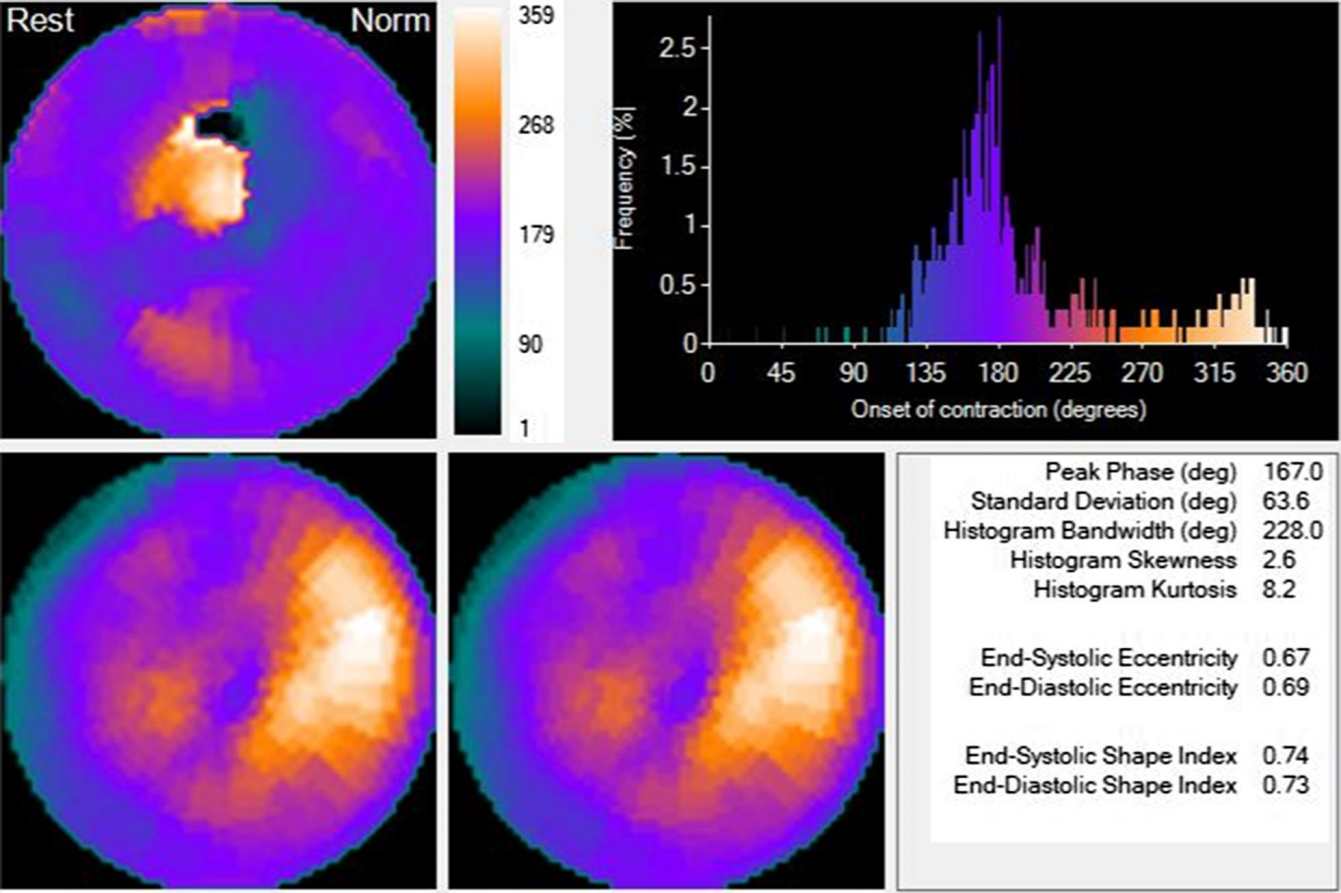
Same patient, intraventricular synchronism behavior pre-CRT. There is a marked dyssynchrony. *CRT* cardiac resynchronization therapy, *HBW* histogram bandwidth, *PSD* phase histogram standard deviation

**Fig. 4:**
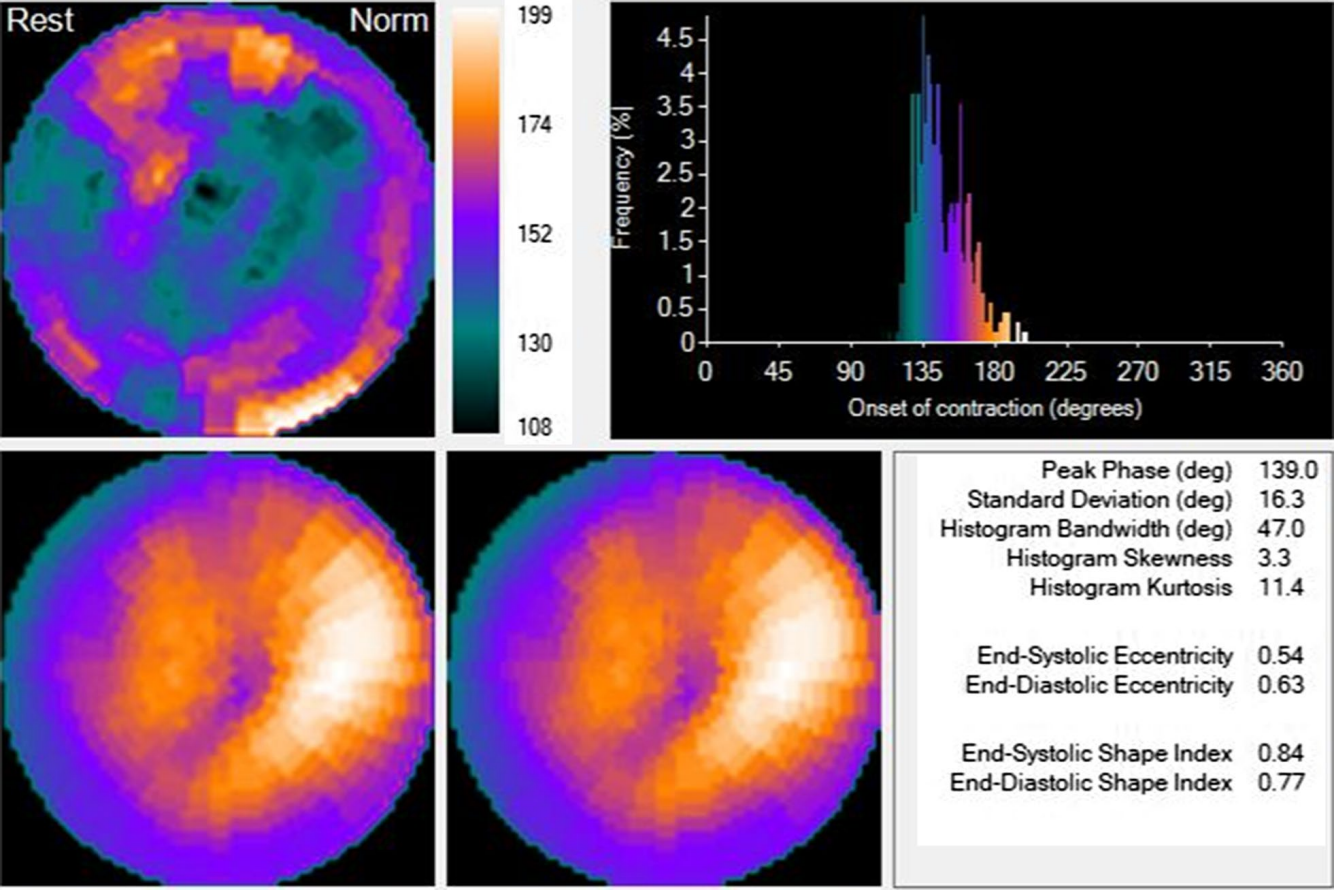
Same patient, intraventricular synchronism behavior post-CRT. Compared to Fig. 3, it shows a significant reduction of dyssynchrony post-CRT.*CRT* cardiac resynchronization therapy, *HBW* histogram bandwidth, *PSD* phase histogram standard deviation

Six months post-CRT, 89% of patients were responders: 11 (100%) of those with non-compaction CM, 25 (86%) of those with ischemic CM, and 47 (89%) of patients with non-ischemic CM. There was no difference according to sex in the number of responders (89% among men versus 88% among women). An example of a responder patient is shown in Figs. 1, 2, 3, and 4.

During the 2 years’ of follow-up, 12 patients (14.9%) died, six (7.4%) were admitted due to HF, and one (1.2%) due to endocarditis. No patient with non-compaction CM had adverse events during the follow-up. Non-ischemic CM showed more HF admissions (5), but ischemic ones showed more deaths (8).

## Discussion

There is a paucity of information regarding CRT implantation in patients with non-compaction cardiomyopathy, and when published, series are generally small. In addition, those published cases with implanted CRT were compared only to DCM and dyssynchrony was evaluated by echocardiography. As far as we know, this is the first work comparing non-compaction CM with ischemic and non-ischemic CM using gated-SPECT phase analysis to evaluate pre- and post-CRT intraventricular dyssynchrony.

In the present study we found that CRT contributes to a marked improvement in non-ischemic CM patients with non-compaction myocardium. We showed a functional improvement in all CM groups, but those more dyssynchronous at baseline (the ones with non-compaction CM) exhibited the more significant improvement on the intraventricular synchronism: PSD was reduced from 89.5 ± 14.2° at baseline to 63.7 ± 20.5° post-CRT (*p* = 0.028). As we previously showed [22], a dyssynchrony reduction as measured by LV PSD could be associated with outcome improvement.

Gated-SPECT MPI contributes to the differentiation between ischemic and non-ischemic ventricular dysfunction. Ischemic dysfunction usually causes diffuse, severe, and extensive perfusion defects and wall motion abnormalities in coronary artery distribution, whereas those with non-ischemic etiology have either homogeneous tracer uptake or mild to moderate perfusion defects in non-coronary artery distribution, as well as diffuse wall motion abnormalities [32, 33]. This coincides with our results, where mean SRS was significantly higher for ischemic patients (14.9 ± 7.1) versus 9 ± 3.9 (non-ischemic) and 8.7 ± 4.4 (non-compaction CM patients), *p* < 0.0001.

Although the quality of life (measured by the MLHFQ) significantly improved in all groups, which could also be mediated by placebo effect in some cases, non-ischemic patients (with or without non-compaction) showed a better functional improvement (objectively measured by LVEF increase and EDV and ESV reduction) at 6 months post-CRT.

The presence of baseline mechanical dyssynchrony measured by echo has been associated with a response to resynchronization therapy [34]. Delgado et al. [14] found that baseline LV radial dyssynchrony, discordant LV lead position, and myocardial scar in the region of the LV pacing lead were independent determinants of long-term prognosis in ischemic HF patients treated with CRT. However, no potential marker of mechanical dyssynchrony reliably predicts response, and interobserver variability is an important drawback to consider, mainly if echocardiography is the imaging technique used. Gated-SPECT phase analysis is a useful and validated option to evaluate mechanical intraventricular dyssynchrony and predict post-CRT results [19, 25, 31, 35]. In our previous work in the IAEA research project [22] all baseline and post-CRT studies were processed in a core laboratory. We found that the difference between baseline and six months post-CRT dyssynchrony is a sensitive parameter of clinical outcomes, rather than the baseline value by itself. Thus, LV dyssynchrony automatically measured by PSD from gated-SPECT MPI is a valid marker of CRT clinical outcomes.

In the present work we have found that CRT caused a reduction of the dyssynchrony measured through PSD and HBW in all groups. Nevertheless, those more dyssynchronous at baseline (the patients with non-compaction CM) exhibited the more significant improvement in the intraventricular synchronism: PSD was reduced from 89.5 ± 14.2° at baseline to 63.7 ± 20.5° post-CRT (*p* = 0.028).

The activation of neurohormonal and cytokine systems during the progression of HF leads to alterations in myocyte biology, myocyte loss, and alterations in extracellular matrix, as well as alterations in LV chamber geometry (LV remodeling) [36]. Thus, it is understandable that treatments which can revert this remodeling (as CRT) will originate a functional improvement, as we found among our cases, mainly in those with non-compaction CM.

Several authors [37–39] reported that SRS was an independent predictor of mechanical dyssynchrony, and this is plausible considering that infarcted and ischemic segments with WM abnormalities lead to an abnormal contraction pattern and dyssynchrony [40]. Compared to non-ischemic ones, our ischemic patients experienced more hard events: Non-ischemic CM showed more heart failure admissions (5), but ischemic ones showed more deaths. Although the difference was not significant (this might be due to the small sample included), the ischemic group showed the lower number of responders (86%) compared to the non-ischemic (89%) and the non-compaction CM (100%). This coincides with other reports, where patients with non-ischemic cardiomyopathy respond more frequently than those with ischemic heart disease [41].

Although it has been reported that females have considerably higher response rates to CRT than males [41], we did not find the same. In fact, in our study there was no difference according to sex in the number of responders (89% among men versus 88% among women) and we do not have a specific explanation for this.

Dyssynchrony and CRT outcome in non-compaction cardiomyopathy.

Left ventricular non-compaction (or left ventricular hypertrabeculation) is a morphological abnormality of the left ventricular myocardium, characterized by a meshwork of myocardial strings, interlacing, and orderless in arrangement, most frequently located in the apex and the lateral wall. LVNC is believed to be congenital in the majority of cases, but may develop during life in single cases (acquired LVNC) [42]. The prevalence of adult LVNC ranges from 0.01% to 0.27% [43, 44], and it is more frequent in men (about two-thirds) than women in the majority of the studies [42, 43, 45].

In LVNC patients, HF is primarily due to systolic dysfunction, and the leading symptom is dyspnea. Other common manifestations include arrhythmias such as ventricular tachycardia and atrial fibrillation, as well as systemic thromboembolic events. The most frequent electrocardiocardiographic abnormality is LBBB, reported in up to 56% of cases [43].

LVNC per se does not require a specific treatment. Adequate therapy is indicated only in case of complications, such as ventricular arrhythmias, cardioembolism, or systolic dysfunction. CRT is indicated in case of intractable HF despite optimal medical treatment, and mechanical dyssynchrony [46]. CRT has been applied in single LVNC patients and has shown a beneficial effect [26–28, 42, 47].

In our work, those patients with non-compaction CM showed the higher improvement both functionally and in terms of reduction of intraventricular dyssynchrony. All of them were responders to CRT. This coincides with the results of Bertini et al. [28]. These authors compared the effects of CRT on LV reverse remodeling in 52 patients with DCM associated or not with isolated LVNC by using standard and contrast echocardiography to assess LV volumes and function and to optimize visualization of the endocardial border at baseline and at 6-month follow-up. They concluded that patients with LVNC and CM had greater LV reverse remodeling after CRT than did patients with DCM. The greater the area of non-compaction (higher number of LVNC segments) the greater the chance of achieving CRT response and greater LV reverse remodeling [28].

It has been reported that dyssynchrony between non-compacted and compacted myocardium contributes to global LV dysfunction [4]. However, Bertini et al. did not find differences in LV dyssynchrony between LVNC CM and DCM patients, although in the subset of LVNC CM patients, CRT achieved greater LV reverse remodeling and determined more super-responders than in patients with DCM [28]. We also analyzed the intraventricular dyssynchrony behavior by using phase analysis gated-SPECT, which constitutes the novelty of our work in comparison with previously published papers on CRT on LVNC patients. In contrast with Bertini’s paper, we did find higher intraventricular dyssynchrony in LVNC patients compared to those with DCM, and its reduction by CRT may contribute to the higher functional improvement observed in these LVNC patients. The fact of considering the higher reproducibility of nuclear measurements compared to other imaging techniques (as echo) may represent an added value in the clinical management of LVNC patients.

Interestingly, Bertini et al. [28] indicated that their data seem in agreement with the hypothesis that LVNC is part of a more widespread cardiomyopathy, involving both the morphologically normal and the dysmorphic segments, and that, in particular, the LVNC segments may represent a phenotypic expression of this disease that may be partially or totally reversible. Indeed, in a single case with neuromuscular disorder, Stöllberger et al. reported a complete regression of LVNC areas after CRT [48]. This represents a thought-provoking phenomenon which should be more thoroughly studied.

On the other hand, it has been reported that LVNC segments have, paradoxically, a better performance than morphologically normal ones [49]. In their work, Bertini et al. hypothesized that, according to this, and taking into account that the LV lead is positioned in a (postero-)lateral vein tributary of LVNC areas in the majority of patients, pacing LVNC segments may provide beneficial effects on LV function, partially explaining the larger percentage of super-responders they found as compared with patients with DCM [28]. In our case, this explanation seems possible as well, because in 82% of our LVNC patients the LV lead was positioned in the lateral wall.

## Limitation

A small sample of patients was included, mainly of those who had a non-compaction CM. Anyway, this condition is not frequent, and we also offer the information regarding the intraventricular dyssynchrony by using gated-SPECT phase analysis, which can be considered as an added value.

### Conclusion

CRT contributes to a marked improvement in non-ischemic CM patients with non-compaction myocardium. Phase analysis in gated-SPECT MPI is a valuable tool to assess the response to CRT.

## Data Availability

All data generated or analyzed during this study are included in this published article.

## References

[1] Najafi F, Jamrozik K, Dobson AJ (2009). Understanding the ‘epidemic of heart failure’: A systematic review of trends in determinants of heart failure. Eur J Heart Fail..

[2] Giubbini R, Milan E, Bertagna F, Mut F, Metra M, Rodella C (2009). Nuclear cardiology and heart failure. Eur J Nucl Med Mol Imaging..

[3] Gheorghiade M, Bonow RO (1998). Chronic heart failure in the United States: a manifestation of coronary artery disease. Circulation.

[4] Lofiego C, Biagini E, Pasquale F, Ferlito M, Rocchi G, Perugini E (2007). Wide spectrum of presentation and variable outcomes of isolated left ventricular non-compaction. Heart.

[5] Lewin M (2010). Left ventricular noncompaction: travelling the road from diagnosis to outcomes. J Am Soc Echocardiogr..

[6] Biagini E, Ragni L, Ferlito M, Pasquale F, Lofiego C, Leone O (2006). Different types of cardiomyopathy associated with isolated ventricular noncompaction. Am J Cardiol..

[7] Jenni R, Rojas J, Oechslin E (1999). Isolated noncompaction of the myocardium. N Engl J Med..

[8] Leclercq C, Kass DA (2002). Retiming the failing heart: Principles and current clinical status of cardiac resynchronization. J Am Coll Cardiol..

[9] Abraham WT, Hayes DL (2003). Cardiac resynchronization therapy for heart failure. Circulation.

[10] Leclercq C, Hare JM (2004). Ventricular resynchronization: Current state of the art. Circulation.

[11] Leclercq C, Faris O, Tunin R, Johnson J, Kato R, Evans F (2002). Systolic improvement and mechanical resynchronization does not require electrical synchrony in the dilated failing heart with left bundle-branch block. Circulation.

[12] Sillanmaki S, Lipponen JA, Tarvainen MP, Laitinen T, Hedman M, Hedman A (2019). Relationships between electrical and mechanical dyssynchrony in patients with left bundle branch block and healthy controls. J. Nucl. Cardiol..

[13] Bax JJ, Bleeker GB, Marwick TH, Molhoek SG, Boersma E, Steendijk P (2004). Left ventricular dyssynchrony predicts response and prognosis after cardiac resynchronization therapy. J Am Coll Cardiol..

[14] Delgado V, van Bommel RJ, Bertini M, Borleffs CJ, Marsan NA, Arnold CT (2011). Relative merits of left ventricular dyssynchrony, left ventricular lead position, and myocardial scar to predict long term survival of ischemic heart failure patients undergoing cardiac resynchronization therapy. Circulation.

[15] Bax JJ, Abraham T, Barold SS, Breithardt OA, Fung JW, Garrigue S (2005). Cardiac resynchronization therapy: Part 1—Issues before device implantation. J Am Coll Cardiol..

[16] Tanaka H, Nesser HJ, Buck T, Oyenuga O, Jánosi RA, Winter S (2010). Dyssynchrony by speckle-tracking echocardiography and response to cardiac resynchronization therapy: Results of the Speckle Tracking and Resynchronization (STAR) study. Eur Heart J..

[17] Leyva F (2017). The role of cardiovascular magnetic resonance in cardiac resynchronization therapy. Heart Fail Clin..

[18] Badhwar N, James J, Hoffmayer KS, O’Connell JW, Green D, De Marco T (2016). Utility of equilibrium radionuclide angiogram-derived measures of dyssynchrony to predict outcomes in heart failure patients undergoing cardiac resynchronization therapy. J Nucl Med..

[19] Chen J, Garcia EV, Folks RD, Cooke CD, Faber TL, Tauxe EL (2005). Onset of left ventricular mechanical contraction as determined by phase analysis of ECG-gated myocardial perfusion SPECT imaging: Development of a diagnostic tool for assessment of cardiac mechanical dyssynchrony. J Nucl Cardiol..

[20] Chen J, Garcia EV, Bax JJ, Iskandrian AE, Borges-Neto S, Soman P (2011). SPECT myocardial perfusion imaging for the assessment of left ventricular mechanical dyssynchrony. J Nucl Cardiol..

[21] Henneman M, Chen J, Dibbets-Schneider P, Stokkel MP, Bleeker GB, Ypenburg C (2007). Can LV dyssynchrony as assessed with phase analysis on gated myocardial perfusion SPECT predict response to CRT?. J Nucl Med..

[22] Peix A, Karthikeyan G, Massardo T, Kalaivani M, Patel C, Pabon LM, et al. Value of intraventricular dyssynchrony assessment by gated-SPECT myocardial perfusion imaging in the management of heart failure patients undergoing cardiac resynchronization therapy (VISION-CRT). J Nucl Cardiol. 2019 Jan 25. doi: 10.1007/s12350-018-01589-5. [Epub ahead of print]. 10.1007/s12350-018-01589-5 PMC7921049 30684258

[23] Patel C, Kalaivani M, Karthikeyan G, Peix A, Kumar A, Massardo T, et al. Effect of cardiac resynchronization therapy on septal perfusion and septal thickening: Association with left ventricular function, reverse remodelling and dyssynchrony. J Nucl Cardiol. 2019. doi: 10.1007/s12350-019-01704-0.[Epub ahead of print]. 10.1007/s12350-019-01704-0 30977094

[24] Alexanderson-Rosas E, Espinola-Zavaleta N, Garcia EV, Peix A, Massardo T, Pabon LM, et al. Diastolic dyssynchrony assessment by gated myocardial perfusion- SPECT in subjects who underwent cardiac resynchronization therapy. J Nucl Cardiol. 2019. doi: 10.1007/s12350-019-01845-2. [Epub ahead of print]. 10.1007/s12350-019-01845-2 31410734

[25] Jimenez-Heffernan A, Butt S, Mesquita CT, Massardo T, Peix A, Kumar A, et al. Technical aspects of gated SPECT MPI assessment of left ventricular dyssynchrony used in the VISION-CRT study. J Nucl Cardiol. 2020 May 11. doi: /10.1007/s12350-020-02122-3. [Epub ahead of print]. 10.1007/s12350-020-02122-3 PMC8249285 32394405

[26] Oginosawa Y, Nogami A, Soejima K, Aonuma K, Kubota S, Sato T (2008). Effect of cardiac resynchronization therapy in isolated ventricular noncompaction in adults: follow-up of four cases. J Cardiovasc Electrophysiol..

[27] Stöllberger C, Blazek G, Bucher E, Finsterer J (2008). Cardiac resynchronization therapy in left ventricular hypertrabeculation/non-compaction and myopathy. Europace..

[28] Bertini M, Ziacchi M, Biffi M, Biagini E, Rocchi G, Martignani C (2011). Effects of cardiac resynchronisation therapy on dilated cardiomyopathy with isolated ventricular non-compaction. Heart.

[29] Jenni R, Oechslin E, Schneider J, Attenhofer Jost C, Kaufmann PA (2001). Echocardiographic and pathoanatomical characteristics of isolated left ventricular non-compaction: a step towards classification as a distinct cardiomyopathy. Heart.

[30] Cerqueira M, Weisman M, Dilsizian V, Jacobs AK, Kaul S, Laskey WK (2002). Standardized myocardial segmentation and nomenclature for tomographic imaging of the heart. Circulation.

[31] Henneman MM, Chen J, Ypenburg C, Dibbets P, Bleeker GB, Boersma E (2007). Phase analysis of gated myocardial perfusion single-photon emission computed tomography compared with tissue Doppler imaging for the assessment of left ventricular dyssynchrony. J Am Coll Cardiol..

[32] Ananthasubramaniam K, Dhar R, Cavalcante JL (2011). Role of multimodality imaging in ischemic and non-ischemic cardiomyopathy. Heart Fail Rev..

[33] Peix A, Karell J, Rodríguez L, Cabrera LO, Padrón K, Carrillo R (2014). Gated SPECT Myocardial Perfusion Imaging, Intraventricular Synchronism, and Cardiac Events in Heart Failure. Clin Nucl Med..

[34] Bleeker GB, Mollema SA, Holman ER, Van de Veire N, Ypenburg C, Boersma E (2007). Left ventricular resynchronization is mandatory for response to cardiac resynchronization therapy: Analysis in patients with echocardiographic evidence of left ventricular dyssynchrony at baseline. Circulation.

[35] Boogers MJ, Chen J, van Bommel RJ, Borleffs CJ, Dibbets-Schneider P, van der Hiel B (2011). Optimal left ventricular lead position assessed with phase analysis on gated myocardial perfusion SPECT. Eur J Nucl Med Mol Imaging..

[36] Mann DL. Pathophysiology of heart failure. In: Bonow R, Mann D, Zipes D, et al, eds. Braunwald’s Heart Disease. A Textbook of Cardiovascular Medicine. Philadelphia, PA: Elsevier Saunders; 2012:487–504.

[37] Samad Z, Atchley AE, Trimble MA, Sun JL, Shaw LK, Pagnanelli R (2011). Prevalence and predictors of mechanical dyssynchrony as defined by phase analysis in patients with left ventricular dysfunction undergoing gated SPECT myocardial perfusion imaging. J Nucl Cardiol..

[38] Bader H, Garrigue S, Lafitte S, Reuter S, Jaïs P, Haïssaguerre M, et al. Intra-left ventricular electromechanical asynchrony. A new independent predictor of severe cardiac events in heart failure patients. J Am Coll Cardiol. 2004;43:248–56. 10.1016/j.jacc.2003.08.038 14736445

[39] Cho GY, Song JK, Park WJ, Han SW, Choi SH, Doo YC (2005). Mechanical dyssynchrony assessed by tissue Doppler imaging is a powerful predictor of mortality in congestive heart failure with normal QRS duration. J Am Coll Cardiol..

[40] Forrester JS, Wyatt HL, Da Luz PL, Tyberg JV, Diamond GA, Swan HJ (1976). Functional significance of regional ischemic contraction abnormalities. Circulation.

[41] Mark Estes NA. Examining Achilles’ Heel. Improving response rates with cardiac resynchronization therapy. J Am Coll Cardiol Img. 2014;7:1249–50. 10.1016/j.jcmg.2014.09.007 25496543

[42] Finsterer J (2010). Left ventricular non-compaction and its cardiac and neurologic implications. Heart Fail Rev..

[43] Oechslin EN, Attenhofer Jost CH, Rojas JR, Kaufmann PA, Jenni R (2000). Long-term follow-up of 34 adults with isolated left ventricular noncompaction: a distinct cardiomyopathy with poor prognosis. J Am Coll Cardiol..

[44] Stöllberger C, Blazek G, Winkler-Dworak M, Finsterer J (2008). Sex differences in left ventricular noncompaction in patients with and without neuromuscular disorders. Rev Esp Cardiol..

[45] Stöllberger C, Finsterer J (2004). Left ventricular hypertrabeculation/noncompaction. J Am Soc Echocardiogr..

[46] Saito K, Ibuki K, Yoshimura N, Hirono K, Watanabe S, Watanabe K (2009). Successful cardiac resynchronization therapy in a 3-year-old girl with isolated left ventricular non-compaction and narrow QRS complex. Circ J..

[47] Garnier A, Girod G (2009). Cardiac resynchronization therapy in a patient with isolated ventricular non-compaction: a case report. Eur J Echocardiogr..

[48] Stöllberger C, Keller H, Finsterer J (2007). Disappearance of left ventricular hypertrabeculation/noncompaction after biventricular pacing in a patient with polyneuropathy. J Card Fail..

[49] Lofiego C, Biagini E, Ferlito M (2006). Paradoxical contributions of non-compacted and compacted segments to global left ventricular dysfunction in isolated left ventricular noncompaction. Am J Cardiol..

